# Adrenal hemorrhage in newborn: how, when and why- from case report to literature review

**DOI:** 10.1186/s13052-019-0651-9

**Published:** 2019-05-08

**Authors:** M. S. Toti, P. Ghirri, A. Bartoli, C. Caputo, E. Laudani, F. Masoni, L. Mele, R. Bernardini

**Affiliations:** 1 Pediatric Unit, San Giuseppe Hospital, Empoli, Florence Italy; 2 Neonatology and Neonatal Intensive Care Unit, Santa Chiara University Hospital, Pisa, Italy

**Keywords:** Neonatal adrenal hemorrhage, Adrenal insufficiency, Ultrasound monitoring, Differential diagnosis, Hormonal therapy

## Abstract

**Background:**

Neonatal adrenal hemorrhage is a relatively uncommon condition (0.2–0.55%). Various risk factors have been reported in addition to birth asphyxia, such as sepsis, coagulation disorders, traumatic delivery, and perinatal injuries. Adrenal hemorrhage usually affects the right adrenal gland (about 70% of cases) while it involves the bilateral adrenal gland only in 10% of cases. In most cases, the event is asymptomatic but, in others, it may be so devastating to determine death by bleeding or adrenal insufficiency.

**Case presentation:**

A case of bilateral neonatal adrenal hemorrhage, with adrenal insufficiency, but with no important risk factors and favorable evolution in a male infant.

**Conclusions:**

This case emphasizes the importance of keeping a non-interventional attitude, avoiding early surgery but carrying out a serial sonographic follow-up. Serial ultrasound monitoring is the most reliable approach during conservative management.

**Electronic supplementary material:**

The online version of this article (10.1186/s13052-019-0651-9) contains supplementary material, which is available to authorized users.

## Background

**Fig. 1:**
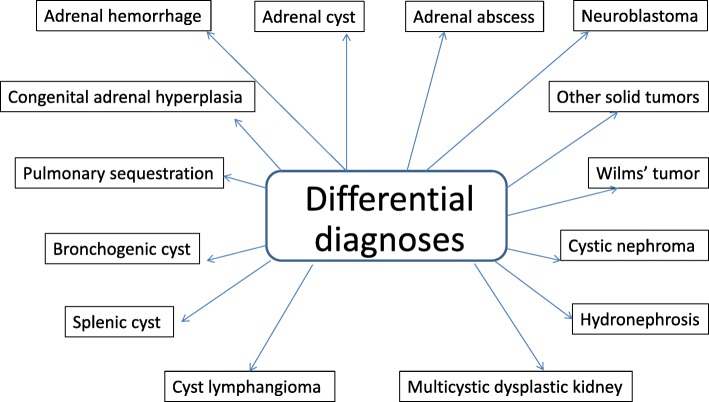
Differential diagnosis of suprarenal abdominal masses in newborn

Neonatal adrenal hemorrhage (NAH) is a relatively uncommon condition (0.2–0.55%) occurring during the neonatal period [1, 2]. In newborns, the large size and increased vascularity of the adrenal glands may result in vulnerability to mechanical compression and sensitivity to changes in venous pressure during delivery [3, 4]. In addition, any factor leading to hypoxia may result in redistribution of blood toward the central nervous system, heart, and adrenal glands [4]. The increased pressure associated with congestion and the damaged endothelial cells associated with hypoxia may cause adrenal hemorrhage [5]. Various risk factors have been reported in addition to birth asphyxia, such as sepsis, coagulation disorders, traumatic delivery, and perinatal injuries. A retrospective study identifies vaginal delivery, macrosomia and fetal acidaemia as the most important risk factors for NAH [6]. However, in a large proportion of cases the etiology of bleeding cannot be established. NAH is most commonly observed in term infants and mainly affects males, perhaps due to the different birth weight [7]. Clinical manifestations are variable or even absent. Sometimes, there are intense jaundice, ischemia, palpable abdominal mass and anemia [8, 9]. Adrenal insufficiency rarely occurs, but when it does it usually appears in the first week of life. In fact, adrenal hemorrhage usually involves the right adrenal gland (about 70% of cases) whereas the bilateral adrenal gland is involved in only 10% of cases. Since the right adrenal gland blood flow drains directly into the inferior vena cava, it is more frequently affected by venous pressure changes and damage, and may be easily compressed between the liver and coasts [10, 11]. Severe unilateral cases often include a hypovolemic shock, while in bilateral events, signs of hypoadrenocorticism are more evident. Different diagnoses of lesion near or at the adrenal gland include adrenal hemorrhage, adrenal cyst, adrenal abscess, neuroblastoma (NBL) or other solid tumors, congenital adrenal hyperplasia (CAH), pulmonary sequestration, bronchogenic cyst, enteric cyst, splenic cyst and cyst lymphangioma ([12], Fig. 1). Some cysts arising from the upper pole of the kidney may have a similar appearance in the image analysis, including duplication of the renal collecting system, hydronephrosis, multicystic dysplastic kidney, Wilms’ tumor, and cystic nephroma ([13], Fig. 1). However judging the nature of a suprarenal mass is sometimes difficult, especially when there is unusual clinical course. It’s important to highlight that in the literature we find cases in which only a surgical exploration was performed to exclude the possibility of malignancy [4, 9]. The most important issue in suspected bleeding is to differentiate these hemorrhagic lesions from NBL, especially in unilateral cases. The adrenal gland has a considerable regenerative capacity and most NAH are not associated with significant adrenal insufficiency. Rarely, progressive or cystic fibrosis alterations in infants or children determine gradual impairment of glandular function resulting in adrenal insufficiency, although prematurity and severe underlying diseases such as sepsis, DIC, perinatal hypoxia and intraventricular hemorrhage are also potential causes of adrenal insufficiency in these patients [14]. NAH is usually self-limited with resolution and a complete regression of lesions within the period of time that goes from the 20th to the 165th day of life as reported by Postek G. et al. [15]. However, in neonates, ultrasound (US) is the preferred modality for both the initial screening and the follow-up evaluation because it is portable, rapid, sensitive, non-invasive and free from ionizing radiation. Computed tomography (CT) and magnetic resonance (MRI) are useful in confirming the presence of hemorrhage and progression of hemoglobin breakdown, but they usually do not provide additional information [16]. The favorable evolution recommends maintaining an observational/non-interventional approach.

Below a case is presented of bilateral NAH with adrenal insufficiency but with no important risk factors and favorable evolution.

## Case presentation

A male infant was born at term (39 weeks and 5 days of gestation) by spontaneous vaginal delivery following an uneventful pregnancy (no gestational diabetes or other complications). The labor delivery monitoring was without worthy alteration and cordonal arterial and venous hemogasanalysis were good (pH 7.303, Base Excess (BE) 3.1, pH 7.341, BE 3.9). He required no resuscitation at birth and the Apgar score was respectively 8 and 9 at minutes 1 and 5. His birth weight was 3350 g (Appropriate for Gestational Age, AGA, 46° percentile). Vitamin K 1 mg IM was given duly at birth. There was no evidence of trauma. Twenty-four hours after delivery the neonate was hyporeactive and drowsy, and the blood exam showed a slight increasing of C-reactive protein (CRP) and hyperbilirubinemia (BT). An antibiotic intravenous (IV) therapy was started and a 5% glucose solution was immediately administered in continuous infusion due to feeding difficulties, achieving that glycemic stability and arterial pressure were within the limits. Indirect hyperbilirubinemia was developed gradually with phototherapy started on the 1st postnatal day (Direct Coombs Test was positive for AB0 incompatibility). Despite the CRP’s value and the jaundice quickly regressed.

**Fig. 2:**
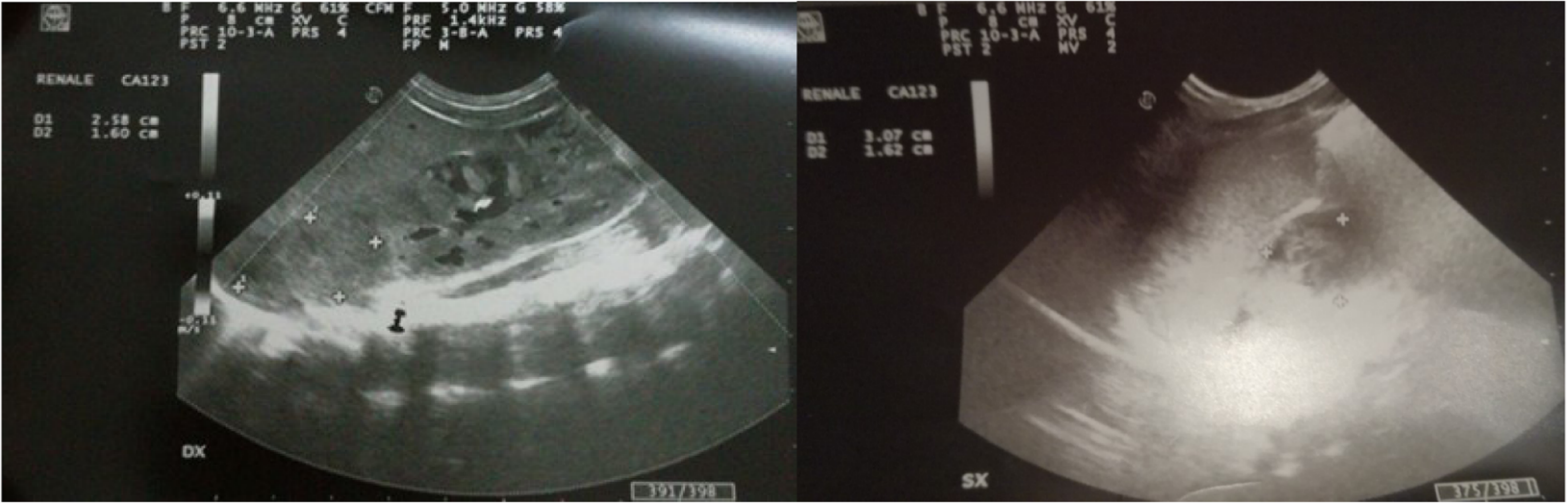
Abdominal ultrasonography revealed suprarenal bilateral lesion, well circumscribed with an inhomogeneous aspect but without vascular flow on Color-Doppler images. The images (25 mm × 16 mm right, 30 × 16 mm left) were mostly isoechoic- hyperechoic. There was a solid portion with essentially normal kidneys and no foci of blood flow within the area

**Fig. 3:**
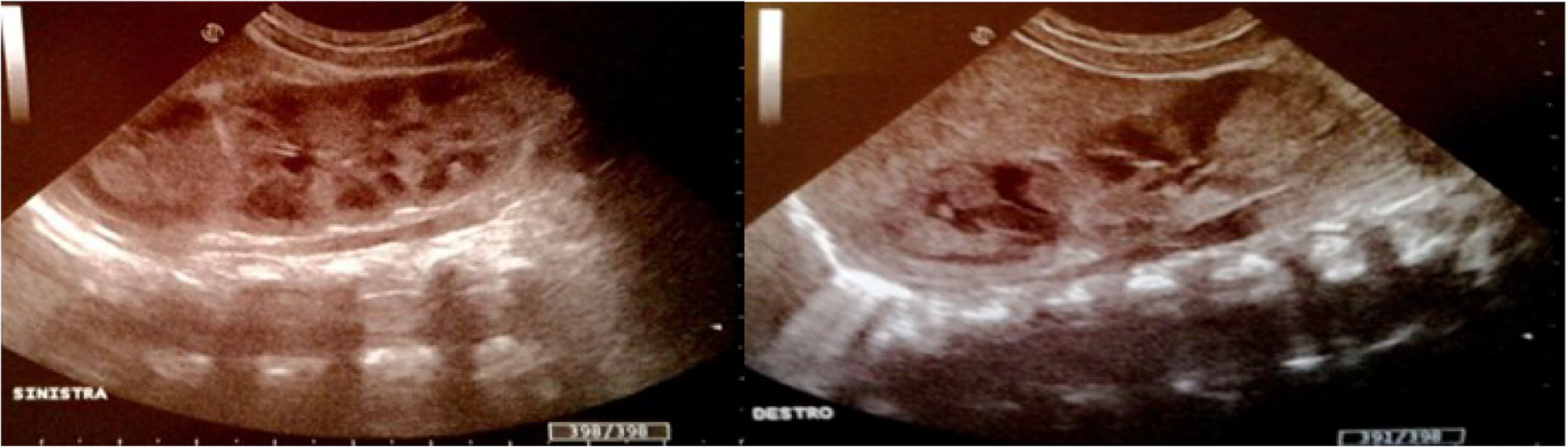
Evolution of the echographic aspect at 15 days of life for modification of the haemorrhagic lesion. The images were mostly isoechoic-anechoic. There was a solid portion, but also fluid level, some internal echoes and minimum turbidity with normal kidneys

At the 10th postnatal day the newborn started to show feeding difficulties, vomiting and electrolytic alteration with hyperkaliemia and hyponatriemia (Table 1). Abdominal ultrasonography revealed suprarenal bilateral lesion, well circumscribed with an inhomogeneous aspect but without vascular flow on color-Doppler images (Fig. 2). The images (25 mm × 16 mm right, 30 × 16 mm left) were mostly isoechoic-anechoic (Figs. 3). There was a solid portion, but also fluid level, some internal echoes and minimum turbidity with essentially normal kidneys and no foci of blood flow within the area of the NAH.

**Table 1:** Blood analysis of the newborn

	I day of life	III days of life	I week of life	III week of life
CRP (mg/dl)	2.3	6.58	0.42	0.01
BT (mg/dl)	11.2 (dir 1,6)	12.5 (dir 0,9)	5.9 (dir 0,6)	1.8
Hb (g/dl)	13.9		10.9	8.4
RBC 106/μ/L	3.87		4.03	2.55
Na mEq/L	140	143	128	138
K mEq/L	4.2	4.4	5.5	4.7
PT			110%	
aPTT			23 s	
AST U/L			39	
ALT U/L			16	
Ferritin ng/ml				32

**Fig. 4:**
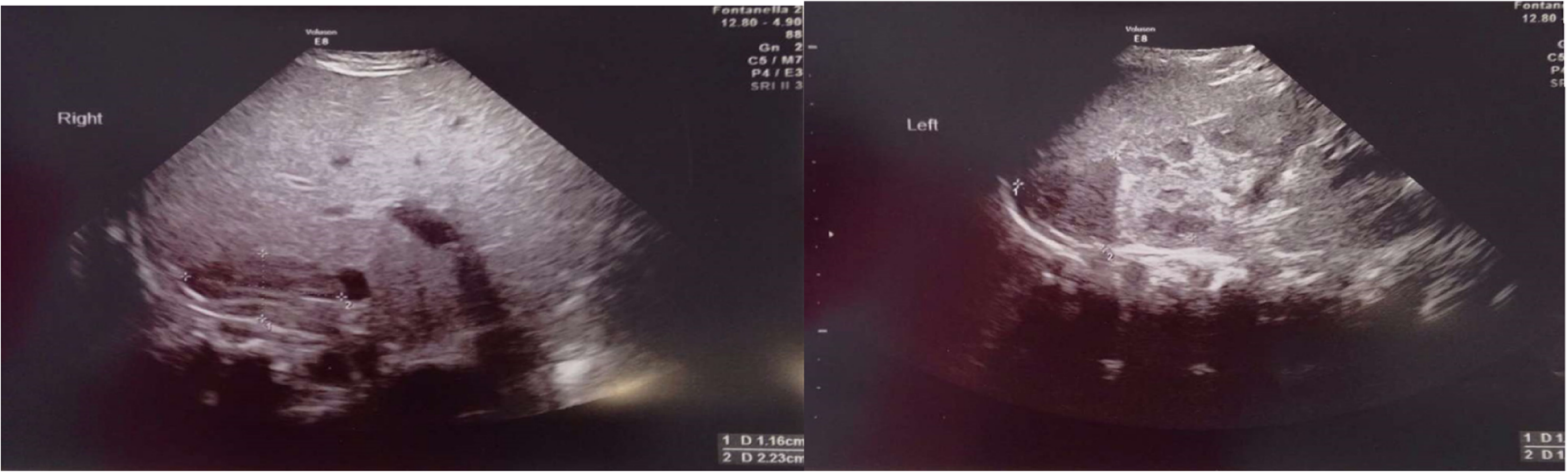
Ultrasound changes of NAH in follow-up with modification of the ultrasonographic appearance

**Fig. 5:**
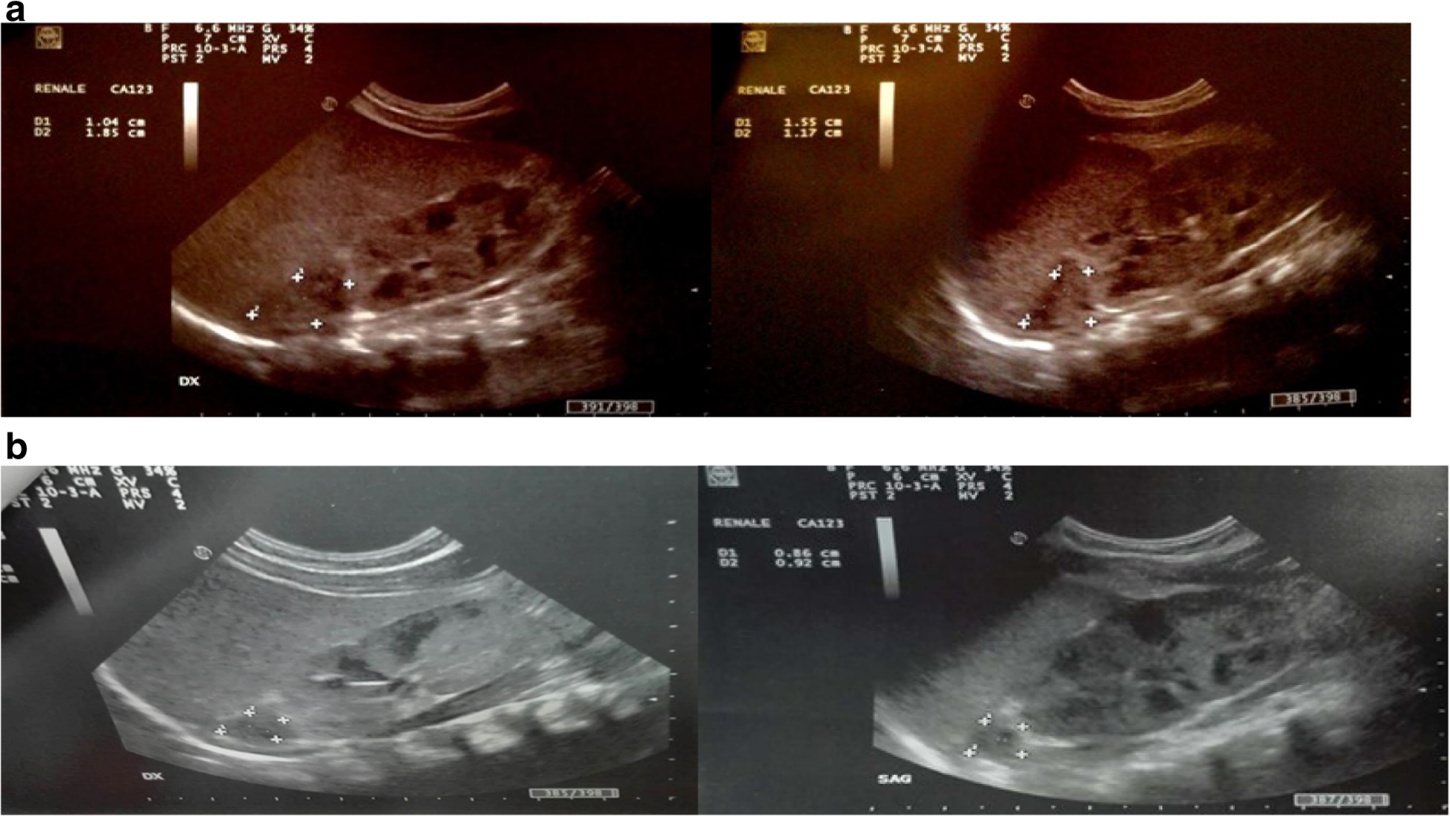
Progressive ultrasound reduction of lesions at 45 days of life (**a**) and at 60 days of life (**b**)

**Fig. 6:**
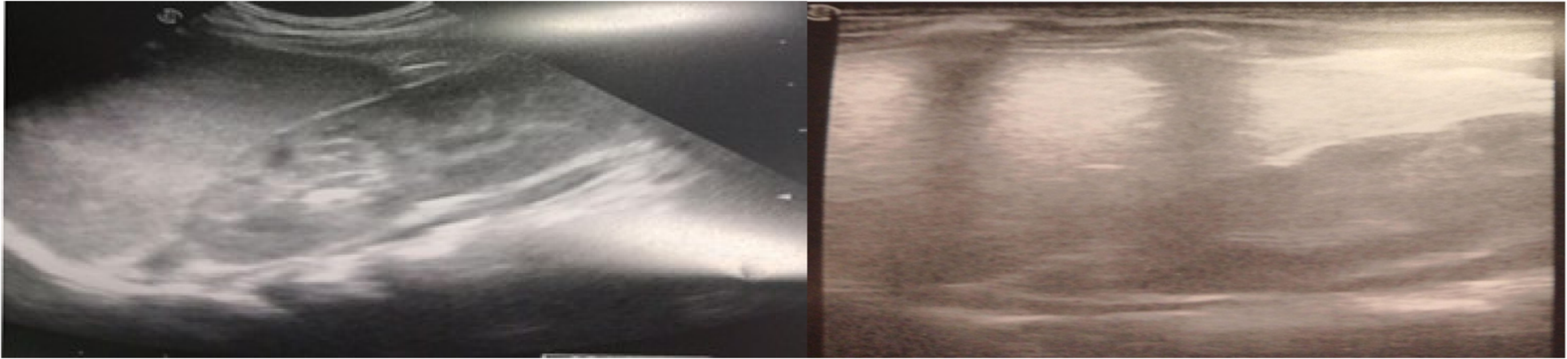
Complete regression of adrenal hemorrhage after 88 days of life with normal renal and adrenal ultrasound appearance

Further investigations revealed normal coagulation profile, no urinary tract infection or other negative blood culture and negative research for invasive neonatal germs (*real-time Polymerase Chain Reaction (PCR)* method), normal urinary catecholamine metabolites, normal 17-OHP value, DHEAS, relationship between plasma renin and aldosterone activity, but high values of ACTH and insufficient cortisol (Table 2). As part of the initial NACL treatment (IV supplementation) and oral hydrocortisone (1 mg/kg) were given; clinical conditions improved rapidly allowing NACL suspension after 3 days. Hormone therapy was reduced progressively with full suspension at the 88th day, allowing no impairment of the hypothalamic-pituitary-adrenal (HPA) axis. Serial ultrasound scans were performed with monthly ambulatory follow-up from the same operator and it was observed a complete regression of lesions after 88 days (Figs. 4, 5, 6 and Additional file 1).

**Table 2:** Steroid hormonal profile, urinary catecholamines, and other analysis of the newborn

	v.n.	1 day of life	10 days of life	14 days of life	30 days of life	45 days of life	60 days of life	88 days of life	110 days of life
17-OH-P ng/ml	0.6–3.3		1.9						
DHEA-solf. μg/ml	0.2–6.9		0.2						
Delta 4 andros. ng/ml	0.3–3.1		0.3						
AVM (mg/24 h)	1–11		0.4						
AOV (mg/g creat.)	0.5–35		17.1						
Noradrenaline ug/24 h			6						
ACTH pg/ml	4.7–49		107.8	149.6		46.4	57.3	24.8	46.8
Cortisol μ/dl	6.7–22.6		6.8	5.3	6.6	3.7	10.4	6.7	9.1
Aldosterone/renin ratio	< 5.2		0.8						
Blood culture		N							
Urine culture			N						

*17-OH-P* 17-hydroxyprogesterone, *DHEA-solf.* Dehydroepiandrosterone sulphate, *Delta 4 andros.* Delta 4 Androstenedione, *AVM* Vanillylmandelic acid, *AOV* Homovanillic acid, *ACTH* Adrenocorticotropic hormone

## Discussion

The cortex and medulla of the adrenal glands have different origins. The medulla originates from neural crest cells of the adjacent sympathetic ganglion, whereas the cortex develops from mesoderm of the posterior abdominal wall. The cortex eventually encircles the medulla. At the end of gestation, the fetal (provisional) cortex accounts for the bulk of the gland. After 7–10 days, the fetal cortex is largely disorganized and necrotic. The narrow peripheral band of cell clusters survives and becomes the source of the permanent cortex [17]. During fetal life, the fetal cortex produces large amounts of precursors of steroid hormones. The placenta utilizes such precursors to produce progesterone to maintain pregnancy and inhibit uterine contractions. The neonatal adrenal gland, unlike in adults, is therefore easy to analyze due to its large size and the reduced amount of retroperitoneal fat. The largest part of the adrenal medulla develops in the neonatal period since fetal catecholamines are produced by Zuckerkland organ and other paraganglia. During fetal life, developing adrenal medulla clusters of small medullary cells are distributed, irregularly, in a highly vascular provisional cortex; when the latter degenerates, the clusters of medullary cells survive and, lacking the support of the cortical cells, aggregate together. The adrenal glands lose about a third of their weight during the first 4 weeks of life, because of the regression of the primitive fetal cortex. With this loss of scaffolding, the medulla coalesces around the central veins. In unselected autopsy cases, the combined average weight of the glands at birth is about 20 g [17]. By the end of the 1st week of life, this mass decreases (as a result of involution of the provisional cortex) to about 12 g, and a further small decrease occurs during the 2nd week of life, such that each gland reaches a weight of approximately 5 g [17]. The overall gland weight remains constant until about the end of the second year of life. After the early neonatal period, the gland appears essentially, very similar to that seen in the adult.

The increased use of perinatal US has led to the detection of an increasing number of suprarenal masses in neonates. In ultrasound images, the adrenal glands have a typical wishbone appearance and form a cap or an inverted V over the kidneys [18]. It is estimated that the prevalence of adrenal incidentaloma is in 5% of radiological case studies and in 2–8% of the autopsy case studies. Thanks to ultrasound, at the beginning, it is possible to mark suprarenal masses highlighting integrity of upper renal pole, then it is possible to follow any changes of the lesion [19].

If there is CAH, it is possible to observe clinically an accumulation of steroidal precursors proximal to the enzyme deficiency, with a resulting diversion into the androgen (therefore male) biosynthesis pathways; this is a recessively inherited condition caused by an enzyme deficiency in the adrenal cortex. In the majority of patients, 21-hydroxylase is the deficient enzyme. This results in virilization in females and early masculinization in males. In the majority of cases, aldosterone cannot be synthesized and some infants may present with severe salt wasting in the neonatal period, which may be fatal. The role of ultrasound in these infants with ambiguous genitalia is to demonstrate the presence of bilateral volumetric increase with cerebriform contours (irregular contours with typically triangular appearance), preserving cortico-adrenal differentiation beyond that of internal female structures (uterus and ovaries). If there is a pulmonary sequestration, it is important to search for a systemic vessel that raises a homogeneous mass.

With NAH, the ultrasound images are different at different stages of bleeding and there is a various spectrum of sonographic appearances. In the early stage, NAH appears solid, enlarged and echogenic. As liquefaction occurs, the mass shows mixed echogenicity with a central hypoechoic region and some internal echoes, and gradually cystic change occurs; this can take 1–2 weeks from the time of hemorrhage. In a short span of time, the hemorrhage starts shrinking and may ultimately be left with a rim of calcification. Sometimes a complex picture may be found due to the coexistence of necrotic tissue, blood clots, calcification and cyst [4]. Calcification may be seen as early as 1–2 weeks after onset. When an adrenal hemorrhage is found, the kidneys must be carefully scanned and measured and the echogenicity evaluated for renal vein thrombosis. The features to look for are, an echogenic enlarged swollen kidney, with the typical increase in echogenicity of the interlobular vessels. Eventually, it resolves completely and becomes anechoic about 2 months later. The majority of neonatal suprarenal masses are identified as congenital neuroblastoma or adrenal hemorrhage. NBL is the most frequent extracranial perinatal malignancy nearly as much as 50% of cases include children aged under 2 years of life [15] and the adrenal gland is the most common primary site [20]. Cancer in newborns is rare, with an estimated incidence of 3.65/100.000 live births and NBL is the most common type of malignant tumor in neonates accounting about 1.75/100.000 of cases [21]. NBL is a poorly differentiated neoplasm derived from neural crest ectoderm [22]. Bilateral involvement of adrenal glands has been noted in less than 10% of cases with NBL and may occur either by synchronous development or metastatic spread of the tumor [23]. Urinary catecholamine metabolites are measured in cases in which a detected mass is suspected to be a NBL. However, catecholamine metabolites are not frequently elevated preoperatively in infants with congenital cystic NBL [24], thus a negative urine catecholamine test cannot exclude the possibility of NBL. The level of urinary catecholamine metabolites does not fulfill the role of a screening test for NBL. However, increased levels of these metabolites may suggest a diagnosis other than NAH. There are two main features of NBL tumors. If they arise within the adrenal gland, they may appear well defined. They may also appear infiltrative, in which case they may have a lobulated outline and there may be small areas of hemorrhage. Lymphadenopathy is often a feature of the tumor mass, and sometimes it may be difficult to differentiate the two. In ultrasound images, the tumor mass appears generally solid in NBL, occasionally with small punctate echogenic areas. Moreover, NBL can be associated by hemorrhaging and, prenatally detected NBL are frequently associated with normal levels of the urinary catecholamine metabolites, which make such differentiations even more difficult [15, 25]. The level of urinary catecholamine metabolites (vanillylmandelic acid) does not fulfill the role of screening test for NBL. However, increased levels of these metabolites may suggest other diagnosis compared to the NAH [26].

Sonographic differentiation between cystic adrenal hemorrhages and cystic NBL may prove to be difficult. Serial US can demonstrate decreases in size and echogenicity, multiloculated cystic mass, calcifications and complete resolution of NAH (14). However, Yamamoto et al. [26] suggested that the regression of the mass screened NBL is possible and not a particularly rare phenomenon. It is therefore important to know the ultrasound response of the various aspects. Color Doppler US examination seems to have most significance in providing a correct diagnosis of adrenal gland hemorrhage. NAH showed no vascular flow in color-Doppler US and gradual regression of lesions over time [27]. In NBL blood supply is essential for its own growth. This tumor gives rise to characteristic high velocity Doppler shifts. In contrast to NBL, NAH is characterized by diminished or absent blood flow [28]. Furthermore Hwang S.M. et al. affirm that serial US follow-up is mandatory for discriminating between cystic congenital adrenal NBL and NAH if conservative management with observation is chosen for a suprarenal cystic lesion in neonates [29].

All adrenal hemorrhages became smaller and more cystic over time. Taking this into consideration, the usual follow-up time for the resolution of the hemorrhages should be within 90 days and NBL should be suspected if the mass is not resolved after 3 months [10]. Besides, the presence of the calcifications on initial images may be indicative of NBL, although calcifications may be a later finding in hemorrhages [10].

## Conclusions

NAH should be suspected even in the absence of important risk factors. In most cases, the event is asymptomatic but it could prove detrimental to determine death by bleeding or adrenal insufficiency. Once established the negativity of the cultures and the absence of risk factors for early-onset sepsis, the CRP increase and the poor clinical conditions in the first day of life may be referred to the unlucky hemorrhagic event. The positive Coombs Test delayed the diagnosis of hemorrhage by losing sight of the real cause of jaundice. Moreover the alteration of the electrolytes and the clinical manifestation are probably attributable to the hormonal insufficiency on the 10th postnatal day. In fact, cytokine-related suppression of ACTH or cortisol synthesis, inadequate perfusion of adrenal gland, limited adrenocortical reserve or immaturity of the hypothalamic-pituitary-adrenal axis may also contribute to the development of adrenal insufficiency. Sometimes it may be necessary to administer hormonal therapy, as shown in this case. Long-term hydrocortisone therapy should not lead to a serious suppressive effect on the later function of the HPA axis. Therefore, it is recommended a rapid suspension of this therapy with clinical or ultrasound improvement. In this case, it is also evident that the first sonographic images showed signs of hemorrhage that occurred previously with an inhomogeneous aspect and an internal echoes such as a progression of hemoglobin changes. It is therefore conceivable that the event may have happened at the birth or immediately later.

However, as the literature affirmed, it is emphasized the importance of keeping a non-interventional attitude is emphasized, avoiding an early surgery approach but carrying out serial sonographic follow-up. US examination is the basic diagnostic modality; when repeated over time, it may allow for monitoring of the evolution of changes and for differentiation with other causes such as malignant and benign tumors. When differentiated from NBL, hemorrhage showed no vascular flow in color Doppler US and gradual regression of lesions overtime. Serial US monitoring is the most reliable method of choice during conservative management. Furthermore, the correct knowledge of the ultrasound patterns allows the safety in the differential diagnosis with other adrenal diseases.

## Additional file

Additional file 1: Patient's timeline. (PDF 177 kb)

## Abbreviations

AGA: Appropriate for Gestational Age
BT: Hyperbilirubinemia
CAH: Congenital adrenal hyperplasia
CRP: C-reactive protein
CT: Computed tomography
HPA: Hypothalamic-pituitary-adrenal
IV: Intravenous
MRI: Magnetic resonance
NAH: Neonatal adrenal hemorrhage
NBL: Neuroblastoma
US: Ultrasound
