# Supplementary material for: Adrenal hemorrhage in newborn: how, when and why- from case report to literature review
Source: Ital J Pediatr. 2019 May 8;45:58. doi: 10.1186/s13052-019-0651-9 (PMC6507044; doi:10.1186/s13052-019-0651-9)
Supplement: Supplementary file 1 — Patient's timeline. (PDF 177 kb) [file 13052_2019_651_MOESM1_ESM.pdf]

Picture 1. Patient Timeline

Male infant was born at term by spontaneous vaginal delivery following an uneventful pregnancy. Apgar score was respectively 8 and 9 at minutes 1 and 5. His birth weight was 3350 g (AGA). Vitamin K 1mg IM was given duly at birth. There was no evidence of trauma. Twenty-four hours after delivery the neonate was hyporeactive and drowsy,

11/2016

Increasing of C-reactive protein and hyperbilirubinemia.

Antibiotic intravenous therapy , 5% glucose solution in continuous infusion and phothery were started immediately with improvement.

12/2016

The baby developed feeding difficulties, vomiting and electrolytic alteration with hyperkaliemia and hyponatriemia, and abdominal distension. Abdominal ultrasonography revealed suprarenal bilateral lesion, well circumscribed with an inhomogeneous aspect but without vascular flow on Color-Doppler images as NAH.

12/2016

Further investigations revealed normal coagulation profile, no urinary tract infection or other, negative blood culture and negative research invasive neonatal germs (*real-time PCR* method), normal urinary catecholamine metabolites, normal 17-OHP value, DHEAS, relationship between plasma renin and aldosterone activity, but high values of ACTH and insufficient cortisol.

NACL treatment for three days and oral hydrocortisone (1 mg/kg) were given so that clinical conditions improved rapidly.

01/2017

F/U 1: Serial ultrasound scans were performed with monthly ambulatory follow-up from the same operator.

02/2017

F/U 2: was observed progressive reduction of lesions with ultrasound.

03/2017

F/U 3: was observed a complete regression of lesions with ultrasound.

03/2017

F/U 4: Hormone therapy was reduced progressively with full suspension at the 88th day allowing no impairment of the hypothalamic-pituitary-adrenal (HPA) axis.

11/2017

Clinical and ultrasound F/U with good auxological growth when the baby's year old. NAH should be suspected even in the absence of important risk factors. Serial ultrasound monitoring is the most reliable method of choice during conservative management. Furthermore, the correct knowledge of the ultrasound patterns allows the safety in the differential diagnosis with other adrenal diseases.

Abbreviations:: AGA(Appropriate for Gestational Age), NAH (neonatal adrenal hemorrhage), F/U (follow up).
